# Glycemic Variability in Type 1 Diabetes Mellitus Pregnancies—Novel Parameters in Predicting Large-for-Gestational-Age Neonates: A Prospective Cohort Study

**DOI:** 10.3390/biomedicines10092175

**Published:** 2022-09-02

**Authors:** Gloria Leksic, Maja Baretić, Lara Gudelj, Marija Radic, Iva Milicic, Marina Ivanišević, Dubravka Jurisic-Erzen

**Affiliations:** 1 Department of Internal Medicine, University Hospital Centre Zagreb, Kispaticeva 12, 10000 Zagreb, Croatia; 2 Division of Endocrinology and Diabetes, Department of Internal Medicine, University Hospital Centre Zagreb, Kispaticeva 12, 10000 Zagreb, Croatia; 3 School of Medicine, University of Zagreb, Salata 3, 10000 Zagreb, Croatia; 4 Department of Internal Medicine, Polyclinic Croatia, Ulica Grada Vukovara 62, 10000 Zagreb, Croatia; 5 Department of Internal Medicine, Clinical Hospital Dubrava, Avenija Gojka Suska 6, 10000 Zagreb, Croatia; 6 Department of Gynaecology and Obstetrics, University Hospital Centre Zagreb, Kispaticeva 12, 10000 Zagreb, Croatia; 7 Department of Endocrinology and Diabetology, University Hospital Centre Rijeka, Kresimirova ulica 42, 51000 Rijeka, Croatia; 8 Faculty of Medicine, University of Rijeka, Ulica Brace Branchetta 20, 51000 Rijeka, Croatia

**Keywords:** diabetes mellitus type 1, pregnancy, large-for-gestational-age neonates, glycemic variability, continuous glucose monitoring, J-index

## Abstract

Pregnancies with type 1 diabetes mellitus (T1DM) have a high incidence of large-for-gestational-age neonates (LGA) despite optimal glycemic control. In recent years, glycemic variability (GV) has emerged as a possible risk factor for LGA, but the results of the conducted studies are unclear. This study analyzed the association between GV and LGA development in pregnancies with T1DM. This was a prospective cohort study of patients with T1DM who used continuous glucose monitoring (CGM) during pregnancy. Patients were followed from the first trimester to birth. GV parameters were calculated for every trimester using the EasyGV calculator. The main outcomes were LGA or no-LGA. Logistic regression analysis was used to assess the association between GV parameters and LGA. In total, 66 patients were included. The incidence of LGA was 36%. The analysis extracted several GV parameters that were significantly associated with the risk of LGA. The J-index was the only significant parameter in every trimester of pregnancy (odds ratios with confidence intervals were 1.33 (1.02, 1.73), 3.18 (1.12, 9.07), and 1.37 (1.03, 1.82), respectively. Increased GV is a risk factor for development of LGA. The J-index is a possible novel GV parameter that may be assessed in all three trimesters of pregnancy together with glycated hemoglobin and time-in-range.

## 1. Introduction

Type 1 diabetes mellitus (T1DM) affects 0.1% of all pregnancies and it is associated with an increased risk for adverse maternal and neonatal outcomes; abortion, preeclampsia, eclampsia, preterm delivery, congenital malformations, large-for-gestational-age neonates (LGA), and macrosomia [1,2,3,4,5].

LGA is one of the most common complications of T1DM in pregnancy, with incidence of 40–60% [1,2,3,4,5]. It is related to many neonatal adverse outcomes, including shoulder dystocia, increased rates of cesarean section, neonatal hypoglycemia, and respiratory distress syndrome; these neonates also become more susceptible to diabetes and obesity later in life [1,2,3,4,5].

So far, studies arguably showed that poor glycemic control in the preconception period and during all trimesters of pregnancy, together with greater gestational weight gain, significantly contribute to fetal overgrowth [1,4,5,6,7,8,9,10,11].

However, pregnancies with T1DM, even with optimal glycemic control and without other risk factors, still have higher LGA incidence compared to type 2 diabetes mellitus (T2DM) patients and gestational diabetes [1]. The reason why is unknown; however, in recent years, many studies have reported about factors “beyond HbA1c” that impact pregnancy outcomes and LGA development [1,2,3]. One of the most described is increased glycemic variability (GV), which is being recognized as a possible risk factor for LGA [1,10,11,12,13,14,15,16,17,18,19,20,21,22,23,24,25]. 

GV is defined by blood glucose fluctuations [26]. Increased GV is associated with higher risk of microvascular and macrovascular diabetic complications [26,27,28,29,30]. 

Nowadays, data from continuous glucose monitoring (CGM) devices are being used to assess GV [27,28]. There are numerous parameters used to describe blood glucose fluctuations and new parameters are constantly arising as CGM becomes widely available, together with a variety of GV calculators [31,32]. The most represented GV parameters in everyday practice are: the percentage coefficient of variation (%CV), the standard deviation of mean glucose (SD), and interquartile range (IQR), which are all easily available from CGM reports [27,28]. Other GV parameters that are less known include continuous overlapping net glycemic action (CONGA), lability index (LI), mean amplitude of glucose excursions (MAGE), mean absolute glucose (MAG), mean of daily differences (MODD), low blood glucose index (LBGI), high blood glucose index (HBGI), average daily risk range (ADRR), M-value, and J-index [31,32,33,34,35,36,37,38,39].

The problem appears in selecting the optimal GV parameter and in defining the target range to evaluate the patient’s GV [40,41]. Although most studies on GV have been conducted on T2DM patients [35,36,37], the same issue persists in T1DM patients, where data on GV are pretty scarce [39,42]. Moreover, GV is an inevitable part of T1DM regardless of glycemic control, and it is much more expressed than in T2DM [1,42]. 

It becomes even more complicated when pregnancy is added to T1DM as the result is a challenging state of hormonal perturbances, insulin resistance, and increasing GV [1,7,8,9]. 

There are few studies on GV in pregnancies with T1DM and they have not achieved a clear consensus on the results; each study has yielded its own GV parameter significantly associated with the LGA risk [1,10,11,12,13,14,15,16,17,18,19,20,21,22,23,24,25]. There is no agreement on when in pregnancy GV should be assessed, which parameters should be used, or when to start an intervention. On the other hand, some studies have not found an association between GV and LGA [20,24]. However, all of these studies had many disadvantages—they were retrospective, some had very small sample sizes, the effects of GV were not the primary endpoints of the studies, various GV parameters were analyzed, and patients used different CGM devices.

Furthermore, it is worth noting that there are very little data about other CGM-derived parameters (time-in-range (TIR), time-above-range (TAR), time-below range (TBR)) and their associations with pregnancy outcomes in T1DM [10,12,16,22,23,25]. As these CGM parameters are emerging as the main therapeutic goals in T1DM [43], it is essential to define their effects on LGA.

Therefore, the primary aim of this study was to analyze GV in T1DM pregnancies and its impact on the development of LGA. The secondary aim was to analyze the association between CGM parameters and LGA.

## 2. Methods

### 2.1. Study Protocol and Study Cohort

This is a prospective cohort study of patients with T1DM who use CGM during pregnancy. The study is being conducted at University Hospital Centre Zagreb, at the state referral center for diabetes in pregnancy. It began in January 2021 and is ongoing. It is registered at ClinicalTrials.gov under number 04997460. ClinicalTrials.gov is a database of privately and publicly funded clinical studies conducted around the world and is a resource provided by the U.S. National Library of Medicine.

The study was approved by the Ethical Committee of the University Hospital Centre Zagreb under number 02/21AG. The included patients signed informed consent.

Patients were included in the study during the first trimester of pregnancy and followed to the birth. Inclusion criteria were ages between 18 and 35 years, body mass index (BMI) < 28 kg/m^2^, glycated hemoglobin (HbA1c) < 8.0%, duration of T1DM > 1 year, CGM device > 3 months, CGM sensor data captured > 70%, signed informed consent.

Excluding criteria were twin pregnancies, HbA1c in the second and third trimester > 8.0%, weight gain > 20 kilograms by the end of the pregnancy, and significant differences in glucose values measured from the CGM device and capillary blood. 

Patients were evaluated every 4–6 weeks when their CGM data were analyzed, together with history, clinical exams, and basic laboratory testing. Patients regularly compared CGM glucose values with capillary blood glucose values on a daily basis.

They all received the same routine multidisciplinary antenatal care, which included a gynecologist, endocrinologist, and diabetic nurse educator.

The preconception patient data were retrieved from medical records; they included age at conception, duration of T1DM, HbA1c, BMI, chronic diabetes complications, autoimmune thyroid disease, and insulin delivery method (multiple daily insulin injection therapy (MDII) or continuous subcutaneous insulin infusion (CSII)).

### 2.2. GV and CGM Data

The same CGM device was used in all participants; intermittently scanned CGM was from the Abbott, FreeStyle Libre Flash Glucose Monitoring System (hereon referred to as CGM). It measures interstitial glucose levels every minute and generates glucose values every 15 min. It consists of a sensor placed subcutaneously and a monitor (reader or smartphone) where the glucose value is presented after scanning the sensor. 

We used the FreeStyle Libre clouding system, Libre View (Abbott), where CGM data are stored and can be shared with the patient’s caregiver. Once in each trimester, CGM data were downloaded to Microsoft Excel where raw glucose data were collected.

CGM data obtained for this analysis included the percentage of time when the sensor was active (sensor data captured), mean glucose, glucose management indicator (GMI), TIR, TBR, TAR, percentage of time spent in very low glucose (<3 mmol/L) and percentage of time spent in very high glucose (>13.9 mmol/L). TIR was set at 3.5–7.8 mmol/L according to the guidelines for CGM and T1DM in pregnancy [43].

EasyGV (available at: https://www.phc.ox.ac.uk/research/resources/easygv, accessed on 30 July 2022) [32] was used to calculate GV parameters once in every trimester from the raw CGM data in Microsoft Excel. EasyGV is an Excel-enabled workbook that is free to use for academic and non-commercial purposes. Additional permission from Oxford University was granted.

Several GV parameters were analyzed for every pregnancy trimester: %CV, SD, CONGA, LI, MAG, MODD, LBGI, HBGI, ADRR, M-value, and J-index. 

%CV shows the magnitude of glucose variability relative to mean blood glucose and SD defines the variation around the mean blood glucose. CONGA is a parameter for differences between a current blood glucose reading and a reading taken hours earlier. LI is a score based on the change in glucose levels over time. MAG is a ratio of absolute differences between sequential glucose readings and the time between the first and last glucose measurement. MODD shows the absolute differences between two glucose values measured at the same time within a 24 h interval. LBGI and HBGI are derived from a logarithmic transformation of the blood glucose scale to render symmetric the skewed distribution of glucose values. ADRR shows the total amount of the daily peak risks for hypo- and hyperglycemia. M-value and J-index are measures of GV as well as parameters of quality of glycemic control. The M-value is derived as a ratio of each glucose value and the total number of glucose values to produce a mean. The J-index is calculated by the formula 0.324 × (mean glucose + SD)^2^ [31,32,33,34,35,36,37,38,39].

GV and CGM parameters are shown for the whole cohort for all trimesters of pregnancy, as well as separately for the LGA and no-LGA pregnancies.

### 2.3. Study Outcomes

The main study outcome was LGA (pregnancies with LGA outcome) or no-LGA (pregnancies without LGA outcome). The other outcomes included birth weight, birth length, birth weight percentile, macrosomia, small-for-gestational-age (SGA) neonates, gestational weight gain, adverse pregnancy outcomes for the mothers (preeclampsia, eclampsia, exacerbation of pre-existing or new-onset retinopathy or nephropathy, new-onset hypertension in pregnancy), and adverse outcomes for the neonate (congenital malformations, preterm delivery, neonatal hypoglycemia, respiratory distress syndrome).

LGA is defined as birth weight > 90th percentile for gestational age and sex, macrosomia is birth weight > 4000 grams, and SGA is birth weight < 6th percentile for gestational age and sex. Preterm delivery is defined as birth before the 37th week of gestation. 

The data on maternal adverse outcomes were collected through regular clinical evaluations and data on neonatal adverse outcomes were retrieved from medical records.

Classification of neonates in the LGA or no-LGA group were performed according to the last available birth percentile charts in Croatia [44].

### 2.4. Statistical Analysis

The normality of the data was analyzed using the Shapiro–Wilk test. The numerical data are presented as median and interquartile range or mean and SD, depending on the distribution of the data. Categorical data are shown as counts and percentages. Differences in GV and CGM parameters between the LGA and no-LGA groups were analyzed using the Mann–Whitney U test. The Wilcoxon signed ranks test was used for the comparison of GV and CGM parameters between the first and third trimesters of pregnancy.

Binary logistic regression (stepwise) analysis was used for testing associations between GV parameters, CGM parameters, and LGA outcomes for each pregnancy trimester. The results are shown as odds ratio (OR) with 95% confidence intervals (CI) and significance (*p*-value) for each variable in the model.

A two-sided *p*-value < 0.05 was assumed to be statistically significant. IBM SPSS Statistics version 24.0 for Windows was used for the analysis.

## 3. Results

In the period from January 2021 to March 2022, there were 107 pregnancies with T1DM; 2.8% (3/107) had spontaneous abortions, 5.6% (6/107) did not want to participate in the study, 6.5% (7/107) used another CGM device besides the Libre Flash CGM, 1.9% (2/107) were excluded due to twin pregnancies, 2.8% (3/107) were excluded based on high HbA1c in the second and third trimesters, 18.7% (20/107) are still pregnant. 

In total, 66 patients were eligible for this analysis.

Patient’s characteristics were as follows: age 30 ± 6 years, BMI 23.9 ± 4.1 kg/m^2^, HbA1c 6.7 ± 1.2%, duration of T1DM 15 ± 9 years. Chronic complications of T1DM were present in 15% (10/66) of patients and included diabetic retinopathy, neuropathy, and nephropathy. A total of 36.4% (24/66) of patients had autoimmune thyroid disease; the median thyroid stimulating hormone (TSH) in the first trimester was 1.25 (IQR 0.8–1.8). MDII was used in 83% of patients and CSII in 17% of patients.

### 3.1. Pregnancy Outcomes

Pregnancy outcomes were as follows: incidence of LGA was 36.4% (24/66), birth weight was 3399 ± 680 grams, birth length 49 ± 3 cm, average week of delivery 37(±2) +3(±2) weeks, and birth weight percentile 66.2 ± 27.6. A total of 16.6% (11/66) of neonates had macrosomia and gestational weight gain was 13 ± 5 kilograms (Table 1). A total of 3% (2/66) of neonates were SGA. A total of 47% (31/66) of neonates were males and 53% (35/66) were females.

A total of 23% (15/66) of patients had adverse pregnancy outcomes: 8% (5/66) of women had new-onset hypertension in pregnancy, 3% (2/66) had preeclampsia, 3% (2/66) had exacerbation of nephropathy, and 1% (1/66) had new-onset retinopathy. There were 6% (4/66) preterm deliveries and 1% (1/66) of infants had neonatal respiratory distress syndrome. There were no congenital malformations or neonatal hypoglycemia.

### 3.2. GV Parameters and LGA

GV parameters were the highest in the first trimester and decreased towards the third trimester of pregnancy (Table 2). This decrease of GV parameters between the first and third trimester was statistically significant for all the parameters except for CONGA (*p* = 0.33); %CV (*p* < 0.01), SD (*p* < 0.01), LI (<0.01), MAG (*p* < 0.01), MODD (*p* < 0.01), HBGI (*p* < 0.01), LBGI (*p* < 0.01), ADRR (*p* < 0.01), M-value (*p* < 0.01), and J-index (*p* < 0.01).

There were differences in the GV parameters between the LGA and no-LGA group, where they were altogether lower in the latter. Yet, statistically significant differences were reached only for the first-trimester SD, CONGA, LI, HBGI, ADRR, J-index, and for the third-trimester CONGA, ADRR, and J-index. There were no statistically significant differences in the second-trimester GV parameters between the LGA and no-LGA group (Table 2).

The logistic regression analysis (stepwise) extracted GV parameters that were significantly associated with the risk of LGA; first-trimester J-index, second-trimester J-index, M-value, %CV and third-trimester J-index, HBGI, ADRR (Table 3).

### 3.3. CGM Parameters

In total, there were 1,019,165 glucose measurements available for the analysis from CGM data; 447,372 glucose measurements for the first trimester, 279,856 and 291,937 for the second and third trimester, respectively.

The mean glucose, GMI, TAR, TBR, time spent in very low glucose, and time spent in very high glucose decreased while TIR increased from the first to the third trimester. These changes were statistically significant for all parameters except for TBR (*p* = 0.89) and very low glucose (*p* = 0.75); GMI (*p* < 0.01), mean glucose (*p* < 0.01), TAR (*p* < 0.01), TIR (*p* < 0.01), very high glucose (*p* < 0.01) (Table 4).

There were differences in CGM parameters between the LGA and no-LGA group in all trimesters, with altogether lower values in the latter. However statistical significance was reached only for the second-trimester TAR and third-trimester GMI, mean glucose, TAR, and TBR. There were no statistically significant differences in the CGM parameters between the LGA and no-LGA group in the second trimester (Table 4).

The logistic regression (stepwise) analysis extracted CGM parameters significantly associated with the risk of LGA; second-trimester TBR (OR = 0.93, CI = 0.87, 1.00, *p* = 0.04), and third-trimester TBR (OR = 0.88, CI = 0.81, 0.97, *p* = 0.01).

## 4. Discussion

This study analyzed GV in pregnancies with T1DM and its association with LGA.

In our cohort, the incidence of LGA was 36%, which is higher than expected considering that there were strict ‘including’ criteria regarding possible risk factors for LGA. However, this higher incidence was still in accordance with other studies [1,10,17,20,22,23].

### 4.1. Glycemic Variability and the Risk for LGA

The data on GV and LGA in pregnancies with T1DM are scarce and unclear as the studies reported conflicting results [1,10,11,12,13,14,15,16,17,18,19,20,21,22,23,24,25]. They have not achieved consensus on pregnancy trimester that is most important for fetal overgrowth and they have not concluded on GV parameters that should be used in the evaluation of GV during pregnancy. 

Some studies suggested that GV early in pregnancy is the most significant risk factor for LGA and that later improvements in glucose control cannot reverse the initial stimulus for fetal overgrowth [15,25,45,46]. According to these, GRADE (glycemic risk assessment diabetic equation), ADRR, SD, LI, and MAGE in the first trimester are related to the risk of LGA [17,21,22].

However, others showed that increased GV in the second and third trimester is the key driver for LGA development [10,11,13,17] and have extracted the J-index, SD, MAGE, HBGI, LBGI, and %CV as GV parameters associated with fetal overgrowth. Nevertheless, there were studies reporting no association between GV and LGA [20,24]. 

Our results confirm that GV in T1DM pregnancy is a significant risk factor for LGA. We demonstrated the importance of GV in all three trimesters of pregnancy, not only in one of the trimesters (which is novel to earlier studies).

The substantial effect of GV in the process of fetal overgrowth is also supported by earlier reports showing that glucose excursions have greater impact on LGA development than chronic hyperglycemia per se [1,45,46,47]. Namely, it is well-known that fetal hyperinsulinemia is the main driver of fetal overgrowth [45,46,47], whereas, for fetal insulin secretion was shown that it is possibly suppressed by chronic hyperglycemia and stimulated by short glucose fluctuations [45,46]. 

This analysis provided answers on GV parameters that are significantly associated with the risk of LGA (Table 3); first-trimester J-index, second-trimester J-index, M-value, %CV and third-trimester J-index, ADRR, HBGI.

J-index was the only significant parameter in all three trimesters of pregnancy. It is a simple measure of GV calculated from the mean glucose and SD using a standardized formula [26].

So far, only one study reported about the J-index in the second trimester as a possible risk factor for LGA [11] while Law et al. [17] showed that the J-index in the first trimester was significantly lower in the LGA group than in the no-LGA group. This is in contrast with our results, which demonstrated a significantly higher first-trimester J-index in LGA pregnancies (Table 2). 

The main issue with the J-index, as well as with most GV parameters, is an unknown cut-off value, which would imply high or low GV. McGrath et al. [11] reported that patients with a J-index > 30 are more likely to have LGA infants. However, this study was conducted on a very small sample size and these results are quite limited for generalization. Our cohort demonstrated the highest J-index in the first-trimester LGA group (median value 36) while the second and third-trimester J-indices were below 30 in the LGA groups. Therefore, we would rather propose setting the J-index cut-off value below 30 when associating with the risk for LGA. 

Moreover, El-Laboudi et al. [42] demonstrated that non-pregnant patients with T1DM have mean J-index of 52.7 (CI = 50.8, 54.6) while healthy controls have mean J-index of 14.3 (CI = 13.2, 15.4). In comparison to this general T1DM population, our cohort had a significantly lower J-index during the whole pregnancy, yet it was still not at the level of a healthy individual and it contributed to fetal overgrowth. 

Furthermore, in the second trimester, %CV was extracted as a significant parameter associated with the risk of LGA, which is a novelty because in earlier studies %CV did not reach statistical significance [10,20]. The advantage of %CV is its availability on every CGM report, so including it in the evaluation in the second trimester does not require any additional calculations. Moreover, in contrast with the other GV parameters, its cut-off is well established at 36%; patients with %CV < 36% have acceptable GV, and %CV > 36% implies high GV [28]. However, there is still some vagueness left regarding %CV. Namely, our cohort demonstrated a slightly higher %CV in the no-LGA group than in the LGA group across all trimesters of pregnancy. Moreover, there is the issue as to whether the well-defined target value <36% applies to pregnant patients with T1DM as the third-trimester %CV in our LGA group was below 36 (median value 32.5).

In the third trimester, together with J-index and ADRR, HBGI was significantly associated with the risk of LGA. This supports the results from former studies [14,47] that demonstrated how third-trimester peaks in glucose especially contribute to fetal overgrowth, even more than chronic hyperglycemia itself.

Furthermore, we observed that GV parameters differed between the LGA and no-LGA group, with altogether lower values in the latter (Table 2). Still, statistically significant differences between groups were achieved only for GV parameters in the first and third trimester. This may imply that second-trimester GV has a smaller contribution to fetal overgrowth compared to the other two trimesters. Notable difference in the first-trimester GV parameters between groups may additionally support the impact of early pregnancy GV on fetal overgrowth, which is in accordance with earlier studies [46]. It was shown that once fetal hyperinsulinemia was established, it could not be reversed despite later changes in the glycemic environment [25,46]. 

Previous studies also reported similar results, with higher GV parameters in pregnancies with LGA outcomes [10,17].

In all, it should be noted that our cohort had lower values of GV parameters when compared with the average population of T1DM [11,28,32,38,42]. For instance, none of our patients had a high-risk ADRR (>40) [38], MODD was <3.3 mmol/L [28] for every trimester and group, J-index was far below 50 [42]. Even so, GV parameters were altogether higher than in the healthy population [32,42] and, despite also attaining good glycemic control, our patients experienced adverse pregnancy outcomes and high rates of LGA.

This may suggest that in pregnancies with T1DM, setting the target value of GV parameters to some calculated number still does not ensure a desirable outcome. Therefore, we propose that the target range of GV markers in these patients be “as low as possible” without aiming for any specific cut-off values.

### 4.2. Continuous Glucose Monitoring Parameters and LGA

So far, studies have shown that achieving higher TIR in all trimesters of pregnancy and less TAR in the second and third trimester, significantly reduces the risk of having LGA [10,12,16,25].

In contrast to these reports, our results demonstrated that TBR in the second and third trimester was a more significant predictor of LGA than TIR and TAR, which did not reach statistical significance. According to these findings, we would suggest maintaining glycemia in the lower part of TIR in the second and third trimester, but certainly still avoiding hypoglycemia.

Furthermore, we observed differences in CGM parameters between the LGA and no-LGA pregnancies, but statistical significance was reached only in the third trimester (Table 4). There was a difference in TAR and TBR but not in TIR, in contrast to the other studies that reported a difference in TIR between the two groups [10,12,25]. A possible explanation lies in the fact that our cohort had well-controlled glycemia during the whole pregnancy, highlighting glucose excursions even more as a significant step in fetal overgrowth.

### 4.3. Strengths and Limitations of the Study

The strength of this analysis is that this is the first prospective study to analyze GV throughout the entire T1DM pregnancies as well as its effect on the development of LGA. Our patients wore CGM devices during all three trimesters and sensor data captured was > 70% for every participant and every trimester. All participants used the same CGM device and CGM data were analyzed by only one physician, which additionally reduced the risk for bias. The study had strict including and excluding criteria and therefore, secluding possible confounding factors for development of LGA except for GV. We used a standardized calculator for GV parameters in contrast to most other studies. Furthermore, to our knowledge, this is the first study to compare GV parameters between pregnant T1DM patients and the general T1DM population and healthy controls.

We also included patients with different methods of insulin delivery, which additionally made it easier for the results to apply to the general T1DM pregnant population. Still, the ratio of MDII to CSII users was significantly in favor of MDII, which can be a barrier.

Limitations include the relatively small sample size and the observational model of the study, which are precluded from the demonstrating causation, but we could only show the association between the possible risk and outcome. As our cohort had strict including and excluding criteria, this could be a limitation as we cannot show the real-world results. Furthermore, there were possible non-respondent biases for patients who dropped out of the study.

In the second and third trimester, we extracted M-value, HBGI, and ADRR as factors significantly associated with the risk of LGA and these GV parameters cannot be easily calculated from CGM data. Therefore, this part of the results is hardly applicable in everyday clinical practice. However, it emphasizes the relevance of GV.

Further studies are needed to confirm these results.

## 5. Conclusions

This study confirmed that increased GV in T1DM pregnancy plays a significant role in the development of LGA. We revealed novel GV parameters as important predictors of fetal overgrowth. We suggest using the J-index as a GV parameter in all trimesters of pregnancy, together with easy obtainable %CV. In the future, the J-index maybe should become a part of regular CGM reports as a GV marker. 

The target range for this marker should be “as low as possible”. The assessment of GV should begin in the first trimester of pregnancy, equally with the evaluation of TIR and HbA1c. Furthermore, we emphasize the need to prevent high glucose excursions in the third trimester as it carries additional risk for fetal overgrowth.

In all, this analysis demonstrates the importance of including GV as a regular part of evaluations in diabetic pregnancies.

We suggest that interventions for reducing GV should begin as early as possible in pregnancy.

## Figures and Tables

**Table 1:** Pregnancy outcomes for the whole cohort, LGA and no-LGA group.

Pregnancy Outcomes	Total	LGA	No-LGA
LGA/%	36.4	/	/
Birth percentile	66.2 ± 27.6	92.4 ± 8.0	51.1 ± 23.2
Birth weight/grams	3399 ± 680	4029 ± 385	3040 ± 535
Birth length/centimeters	49 ± 3	50.5 ± 1.4	47.7 ± 2.5
Week of delivery	37(±2) + 3(±2)	37(±0.8) + 3(±1.9)	36(±1.9) + 2(±2.0)
Macrosomia/%	17	100%	0%
Gestational weight gain/kilograms	13 ± 5	13.4 ± 4.3	12.1 ± 5.7
Maternal adverse outcomes	15% (10/66)	70%	30%
Neonatal adverse outcomes	8% (5/66)	60%	40%

Numerical data are shown as mean and standard deviation. Categorical data are shown as counts and percentages. Legend: LGA (large-for-gestational-age neonates), no-LGA (pregnancies without LGA outcome), SGA (small-for-gestational-age neonates).

**Table 2:** GV parameters in the first, second, and third trimester of pregnancy. GV parameters are shown for the whole cohort and separately for the LGA and no-LGA group. The data are presented as median and interquartile range and the difference in GV parameters between the LGA and no-LGA group was tested with the Mann–Whitney U test (*p*-value). Statistically significant results are bolded.

GV Parameters	Total	LGA	no-LGA	*p*-Value
**First trimester**				
%CV	40.5 (35.9–44.8)	39.0 (37.2–42.6)	42.3 (34.3–45.9)	0.71
SD	2.7 (2.3–3.2)	3.0 (2.6–3.3)	2.5 (2.2–3.1)	**0.01**
CONGA	5.1 (4.2–5.5)	5.5 (5.1–6.5)	4.6 (4.1–5.3)	**<0.01**
LI	12.1 (8.8–17.3)	14.9 (11.0–19.3)	10.4 (8.1–16.1)	**0.02**
MAG	6.3 (5.5–7.2)	6.9 (6.1–8.0)	6.2 (5.4–7.0)	0.11
MODD	2.7 (2.4–3.5)	3.1 (2.6–3.7)	2.5 (2.2–3.4)	0.01
HBGI	5.2 (3.9–7.8)	7.1 (5.2–9.8)	4.6 (3.6–6.8)	**<0.01**
LBGI	6.5 (4.5–9.1)	5.0 (4.0–7.6)	7.9 (4.7–9.2)	0.11
ADRR	23.5 (18.0–31.4)	28.4 (23.5–38.5)	21.6 (16.3–30.0)	**<0.01**
M-value	11.3 (7.3–16.1)	9.5 (7.5–16.3)	13.3 (6.4–16.3)	0.77
J-index	29.8 (22.9–39.0)	36.3 (30.1–47.6)	26.5 (20.7–35.5)	**<0.01**
**Second trimester **				
%CV	37.9 (34.7–43.0)	37.5 (36.0–40.0)	38.9 (33.1–43.4)	0.69
SD	2.3 (2.0–2.8)	2.5 (2.2–2.8)	2.2 (1.8–2.8)	0.23
CONGA	4.6 (4.2–5.3)	4.7(4.3–5.3)	4.5 (3.8–5.3)	0.35
LI	8.4 (6.3–12.6)	9.3 (7.9–12.8)	7.9 (6.0–12.2)	0.21
MAG	5.5 (4.8–6.2)	5.6 (5.0–6.4)	5.2 (4.5–6.2)	0.33
MODD	2.4 (2.1–3.1)	2.6 (2.3–3.1)	2.3 (2.0–3.1)	0.22
HBGI	3.9 (2.7–5.9)	4.2 (3.1–6.3)	3.5 (2.5–5.4)	0.31
LBGI	6.4 (4.4–9.4)	6.0 (4.6–7.9)	7.0 (4.2–9.8)	0.69
ADRR	18.2 (13.3–26.2)	20.2 (14.0–27.0)	17.4 (11.7–23.2)	0.26
M-value	9.5 (6.4–14.8)	8.4 (7.1–11.4)	10.7 (5.4–17.6)	0.51
J-index	24.1 (18.6–33.3)	24.5 (21.3–34.3)	23.6 (16.6–31.4)	0.18
**Third trimester **				
%CV	33.2 (29.5–38.3)	32.5 (29.4–35.7)	34.1 (29.5–39.7)	0.50
SD	2.1 (1.7–2.5)	2.1 (1.9–2.6)	2.0 (1.6–2.4)	0.26
CONGA	4.7 (4.3–5.4)	5.1 (4.4–5.8)	4.5 (4.1–5.4)	**0.04**
LI	7.0 (4.7–8.9)	8.2 (6.5–10.7)	5.9 (4.3–8.8)	0.81
MAG	4.6 (4.0–5.3)	4.8 (4.5–5.3)	4.4 (3.7–5.1)	0.11
MODD	2.1 (1.8–2.6)	2.3 (2.1–2.8)	2.0 (1.7–2.5)	0.06
HBGI	3.4 (2.2–4.9)	3.4 (2.9–6.0)	3.3 (1.8–4.6)	0.19
LBGI	4.4 (3.1–7.4)	4.2 (3.0–5.1)	5.0 (3.2–8.8)	0.14
ADRR	15.2 (11.0–19.9)	17.3 (13.9–24.1)	12.3 (7.8–19.8)	**0.04**
M-value	6.5 (4.5–11.1)	6.1 (4.4–9.0)	8.1 (4.4–15.9)	0.12
J-index	24.0 (17.8–29.3)	25.8 (21.3–33.9)	21.1 (17.0–27.4)	**0.04**

Legend: GV (glycemic variability), LGA (large-for-gestational-age neonates), no-LGA (pregnancies without LGA outcome), %CV (percentage coefficient of variation), SD (standard deviation), CONGA (continuous overlapping net glycemic action), LI (lability index), MAG (mean absolute glucose), MODD (mean of daily differences), LBGI (low blood glucose index), HBGI (high blood glucose index), ADRR (average daily risk range).

**Table 3:** GV parameters in the first, second, and third trimester that are significantly associated with the risk of LGA. The results of the binary logistic regression analysis (stepwise); odds ratio (OR), confidence intervals (CI), and significance for each variable (*p*-value).

GV Parameters	*p*-Value	OR (CI)
**First trimester**		
J-index	0.03	1.33 (1.02, 1.73)
**Second trimester**		
J-index	0.03	3.18 (1.12, 9.07)
M-value	0.01	0.52 (0.32, 0.85)
%CV	0.04	3.24 (1.02, 10.27)
**Third trimester**		
J-index	0.02	1.37 (1.03, 1.82)
HBGI	<0.01	1.48 (1.05, 2.09)
ADRR	0.02	1.31 (1.02, 1.67)

Legend: GV (glycemic variability), LGA (large-for-gestational-age neonates), HBGI (high blood glucose index), LBGI (low blood glucose index), %CV (percentage coefficient of variation), ADRR (average daily risk range).

**Table 4:** CGM parameters in the first, second, and third trimester. CGM parameters are shown for the whole cohort and separately for the LGA and no-LGA group. The data are presented as median and interquartile range and the difference in CGM parameters between the LGA and no-LGA group was tested with the Mann–Whitney U test (*p*-value). Statistically significant results are bolded.

CGM Parameters	Total	LGA	no-LGA	*p*-Value
**First trimester**				
GMI/%	6.4 (6.0–6.9)	6.5 (6.0–7.0)	6.3 (5.9–6.7)	0.41
Mean glucose/mmol/L	7.2 (6.2–8.3)	7.5 (6.2–8.7)	6.9 (6.0–7.9)	0.24
TAR/%	36.5 (25.0–48.7)	39.5 (26.5–52.5)	31.5 (23.2–44.5)	0.18
TIR/%	55.0 (44.0–63.0)	53.0 (41.7–59.2)	56.5 (46.0–65.2)	0.26
TBR/%	7.5 (3.2–16.0)	6.5 (3.7–11.5)	8.5 (3.0–18.5)	0.56
Very low glucose/%	3.0 (1.0–8.0)	2.0 (1.0–6.0)	5.5 (1.0–9.0)	0.27
Very high glucose/%	2.0 (1.0–7.5)	3.0 (1.5–7.5)	1.5 (1.0–8.2)	0.50
**Second trimester**				
GMI/%	5.9 (5.7–6.2)	6.0 (5.8–6.3)	5.9 (5.5–6.2)	0.09
Mean glucose/mmol/L	6.0 (5.6–6.7)	6.3 (5.8–7.0)	6.0 (5.1–6.7)	0.09
TAR/%	20 (15.0–31.7)	22.5 (18.7–33.7)	17.5 (9.7–28.2)	**0.04**
TIR/%	64 (54.2–68.7)	64.5 (55.7–67.0)	63.0 (53.7–69.5)	0.98
TBR/%	10.0 (6.0–21.5)	9.5 (6.0–15.0)	14.0 (6.0–24.2)	0.15
Very low glucose/%	5.0 (2.0–9.5)	4.0 (2.0–7.5)	7.0 (1.2–14.0)	0.13
Very high glucose/%	0.0 (0.0–1.0)	1.0 (0.0–1.5)	0.0 (0.0–1.0)	0.36
**Third trimester**				
GMI/%	6.1 (5.6–6.3)	6.3 (5.9–6.6)	5.8 (5.4–6.3)	**<0.01**
Mean glucose/mmol/L	6.6 (5.5–7.1)	7.0 (6.1–7.7)	6.0 (5.2–7.0)	**0.01**
TAR/%	26.0 (10.5–34.5)	26.0 (19.0–47.0)	19.0 (9.0–32.0)	**0.02**
TIR/%	66.0 (56.5–73.5)	66.0 (52.0–74.0)	66.0 (57.0–72.2)	0.81
TBR/%	6.0 (3.0–12.5)	4.0 (3.0–8.0)	10.0 (3.0–21.5)	**0.04**
Very low glucose/%	2.0 (1.0–6.0)	2.0 (1.0–3.0)	3.0 (0.5–11.5)	0.19
Very high glucose/%	0.0 (0.0–1.0)	0.0 (0.0–2.0)	0.0 (0.0–1.0)	0.18

Legend: CGM (continuous glucose monitoring), LGA (large-for-gestational-age neonates), no-LGA (pregnancies without LGA outcome), GMI (glucose management indicator), TAR (time-above-range), TIR (time-in-range), TBR (time-below-range). Very low glucose (time spent in very low glucose) is defined with % of time spent in very low glucose values < 3.0 mmol/L. Very high glucose (time spent in very high glucose) is defined with % of time spent in very high glucose values > 13.9 mmol/L.

## Data Availability

The main data supporting the results of the current study are presented in this paper. The data generated for this study are quite large and there are also unpublished data in the data sets, so they have not been shared publicly. The data are available from the corresponding author upon reasonable request.

## Abbreviations

GV	glycemic variability
T1DM	type 1 diabetes mellitus
T2DM	type 2 diabetes mellitus
CGM	continuous glucose monitoring
LGA	large-for-gestational-age neonates
MDII	multiple daily insulin injection
CSII	continuous subcutaneous insulin infusion
%CV	percentage coefficient of variation (%CV)
SD	standard deviation of mean glucose
CONGA	continuous overlapping net glycemic action
LI	lability index
MAGE	mean amplitude of glucose excursions
MAG	mean absolute glucose
MODD	mean of daily differences
LBGI	low blood glucose index
HBGI	high blood glucose index
ADRR	average daily risk range
GRADE	glycemic risk assessment diabetic equation
